# Addition of Chlorogenic Acid to Human Semen: Effects on Sperm Motility, DNA Integrity, Oxidative Stress, and *Nrf2* Expression

**DOI:** 10.3390/antiox14040382

**Published:** 2025-03-25

**Authors:** Cinzia Signorini, Roberta Corsaro, Giulia Collodel, Robert Maettner, Karl Sterzik, Erwin Strehler, Laura Liguori, Elena Moretti

**Affiliations:** 1 Department of Molecular and Developmental Medicine, University of Siena, 53100 Siena, Italy; cinzia.signorini@unisi.it (C.S.); laura.liguori@student.unisi.it (L.L.); elena.moretti@unisi.it (E.M.); 2 MVZ Next Fertility Ulm GmbH, 89077 Ulm, Germany; robert.maettner@next-fertility.de (R.M.); ksterzik@aol.com (K.S.); erwin.strehler@next-fertility.de (E.S.)

**Keywords:** chlorogenic acid, DNA integrity, F_2_-Isoprostanes, human semen, sperm motility, *Nrf2* expression, oxidative stress

## Abstract

This study evaluated the effects of chlorogenic acid (CGA) on human semen and on oxidative stress (OS) induced in vitro in human spermatozoa. After the treatment of the basal semen with 100 µM CGA, rapid and slow sperm progressive motility were evaluated and seminal F_2_-Isoprostanes (F_2_-IsoPs), a marker of OS, were quantified by ELISA. In a second set of experiments, semen was treated with 100 µM CGA, 1 mM H_2_O_2_ to induce OS, or H_2_O_2_+CGA; untreated samples were used as controls. Then, sperm motility, DNA integrity by the acridine orange test, F_2_-IsoPs and *Nrf2* mRNA expression by RT-PCR were quantified. In CGA-treated specimens, rapid progressive sperm motility was increased (*p* < 0.01) and F_2_-IsoP levels decreased (*p* < 0.001) versus controls. The increase of F_2_-IsoP levels and DNA damage and the decrease of sperm motility after H_2_O_2_ treatment was reversed in the presence of CGA, which upregulated *Nrf2* mRNA expression. These findings contributed to clarifying CGA’s antioxidant activity and highlighted the positive impact of CGA on sperm progressive motility, suggesting also a possible mechanism of action based on the Nrf2 pathway. CGA can be useful during human semen handling procedures in the laboratory and in optimizing the recovery of motile spermatozoa through selection techniques during assisted reproductive technology protocols.

## 1. Introduction

Infertility is a condition affecting around 8–12% of couples in the reproductive age [1,2]. One of the most significant findings in the field of male infertility is the role of oxidative stress (OS) caused by an imbalance between reactive oxygen species (ROS) production and the available antioxidant buffering capacity [3].

In physiological conditions, ROS are essential for sperm motility, hyperactivation, acrosome reaction, and capacitation [4]. An increase in ROS concentration or a decrease of antioxidant levels causes severe damage to sperm motility, DNA, and, in particular, to membrane lipids, triggering a lipid peroxidation (LPO) cascade. Human spermatozoa have membranes rich in polyunsaturated fatty acids (PUFAs) and contain very low amounts of antioxidants due to their reduced cytoplasm [5]; for this reason, they are extremely vulnerable to LPO, which causes the loss of membrane functions and integrity [6,7]. To mitigate the negative effects of ROS on spermatozoa, an antioxidant battery is present in the seminal plasma [5]. However, OS is a common finding in several male reproductive pathologies such as varicocele [8], genitourinary infections [9] and in a high percentage of what is classified as idiopathic infertility [10,11].

Assisted reproductive techniques (ARTs) represent an important resource in the treatment of infertility; however, gamete handling, and, in particular, semen manipulation procedures such as cryopreservation, centrifugation, light exposure, pH variations and temperature make spermatozoa vulnerable to OS [12,13,14]. A strategy to minimize the sperm oxidative insults, which has received considerable attention during the last years, includes antioxidant supplementation of the media used during semen handling [15,16]. Many antioxidants have been used as supplements, including natural extracts and phenolic compounds [16,17].

Among these substances, chlorogenic acid (CGA), present in several foods such as vegetables, fruits, coffee, and tea [18], has gained attention for its antioxidant properties due to its molecular structure that contains five active hydroxyl groups that scavenge free radicals [19]. The protective action of CGA treatment in animal models with induced pathological reproductive conditions is well documented in the literature [20,21,22]. CGA has also been used to supplement the semen of different animal species to mitigate the effect of OS [23] and it has also been applied to frozen–thawed sperm [24].

In addition, CGA exerts a strong antioxidant effect since it, directly or indirectly, promotes the expression and the activation of antioxidant signaling pathways. For example, CGA reduces the expression of Keap1, an important regulator of the cellular OS response, and it activates the most sensitive transcription factor erythroid 2-related factor 2 (Nrf2) signaling pathway [25]. Nrf2, in turn, plays a crucial role in regulating the levels of several endogenous antioxidant enzymes [26] with a key role in the cellular adaptation to OS caused by pathological events in different cell types. Reduced expression of *Nrf2* has been linked to poor sperm quality and diminished fertility potential, highlighting its importance in male reproductive health as a possible biomarker [27].

Recently, some of our group demonstrated the beneficial effect of CGA supplementation on OS induced in vitro in human spermatozoa selected by the swim up procedure (which removes the seminal plasma, rich in antioxidants) and during cryopreservation procedures [14].

This study aimed to investigate the effect of the supplementation of 100 µM CGA on basal human semen samples. First, the samples of 66 consecutive patients were supplemented with CGA to determine its effect on rapid and slow progressive sperm motility and its protective activity against LPO, quantifying F_2_-isoprostanes (F_2_-IsoPs), an OS marker. Then, we analyzed the effect of CGA on selected normozoospermic semen samples in which OS was induced in vitro (1 mM H_2_O_2_). Several endpoints, such as sperm motility, DNA integrity, and F_2_-IsoPs, were evaluated. Finally, a potential mechanism of action based on *Nrf2* mRNA expression was investigated.

## 2. Materials and Methods

### 2.1. Human Semen Samples

Sixty-six consecutive semen samples were obtained from patients (aged 22–36 years) attending the MVZ Next Fertility Ulm GmbH (Ulm, Germany) laboratory for semen analysis and used for step 1. The semen samples of 10 normozoospermic donors (aged 22–33 years) attending the Department of Molecular and Developmental Medicine, University of Siena (Italy), were used for step 2.

The study was carried out according to the Declaration of Helsinki and the protocol was approved by the following Ethics Committees:

- Ethics Committee of the Federal Institute for Drugs and Medical Devices, ID German Clinical Trials Register DKRS00035009 (step 1);
- Ethics Committee of Siena University Hospital, ID CEAVSE 25612 (step 2).

All participants were informed about the study and provided written informed consent before the inclusion in research, agreeing that their semen samples would be only used for scientific purposes.

### 2.2. Semen Analysis

Semen samples were collected by masturbation in sterile containers after 3–5 days of sexual abstinence. The conventional semen analysis was performed after the liquefaction had been achieved at 37 °C for 30 min. Semen volume, pH, sperm concentration, and motility were evaluated according to the recommendations of the sixth edition of the World Health Organization, Manual for the Examination and Processing of Human Semen [28]. The lower reference limit for semen analysis is the 5th centile. Sperm progressive motility was discriminated as rapid progressive motility and slow progressive motility.

### 2.3. Study Design

#### 2.3.1. Step 1: Effect of CGA on Human Sperm Motility and F_2_-Isoprostane Determination

**Figure 1:**
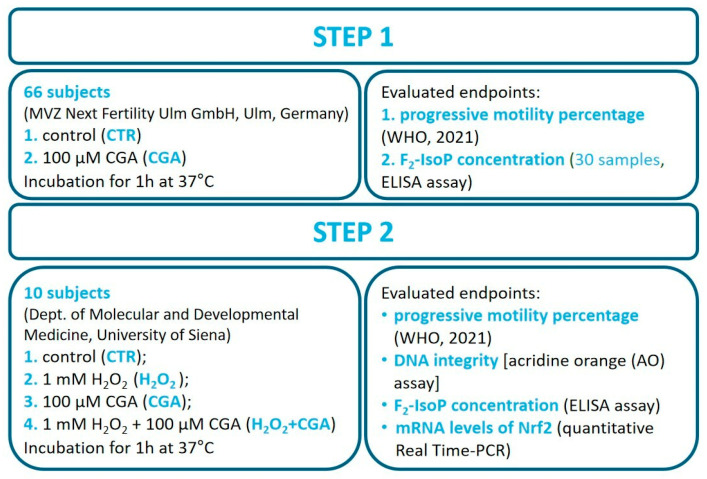
Graphic of the study design illustrating the two steps of the study with the endpoints assessed in each step. Semen analysis was performed following WHO guidelines 2021 [28].

CGA was purchased from Sigma–Aldrich (St. Louis, MO, USA); a stock solution 1 mM was prepared by dissolving the powder in distilled water and stored at 4 °C. After the semen analysis, each of the 66 semen samples were incubated at 37 °C for 1 h with 100 μM CGA (Figure 1). After incubation, sperm motility was evaluated, discriminating rapid and slow progressive motility. Sperm treated in the same conditions but without CGA were used as controls (CTR). In the last 30 samples, F_2_-IsoPs were quantified in the seminal plasma. For this purpose, aliquots were centrifuged at 400× *g* for 15 min and the seminal plasma stored at −80 °C until F_2_-IsoPs were assessed.

##### F_2_-Isoprostane Determination by ELISA Assay

F_2_-isoprostanes were detected by the determination of 8-iso prostaglandin F_2α_ (8-iso PGF_2α_, hereafter referred to 8-isoprostane) in seminal plasma using an ELISA kit (8-Isoprostane ELISA Kit, Cayman Chemical, Ann Arbor, MI, USA). This ELISA kit applies the competitive enzyme immunoassay technique using an anti-8-isoprostane antibody, an 8-isoprostane-AChE tracer, and Ellman’s reagent. The capture antibody (mouse anti-rabbit IgG) was pre-coated onto 96-well plates. The plate was read spectrophotometrically at a wavelength of 405 nm; the concentration of the target molecule in each sample was determined referring to the standard curve (8-Isoprostane standard solutions ranging from 0.8 to 500 pg/mL). The 8-Isoprostane measure was performed in duplicate for each sample and the results were expressed as pg/mL.

#### 2.3.2. Step 2: Effects of CGA on Human Sperm Motility, DNA Integrity, Seminal F_2_-Isoprostane Levels, and *Nrf2* mRNA Expression in Samples Treated In Vitro with 1 mM H_2_O_2_

From a solution of 1 M H_2_O_2_, a concentration of 100 mM was obtained by diluting it 1:10 with distilled water and then used it 1:100 with human semen.

Ten semen samples from normozoospermic donors were divided in 4 aliquots treated as follows:

- untreated sample as control (CTR);
- sample treated with 1 mM H_2_O_2_ to induce OS (H_2_O_2_);
- sample treated with 100 µM CGA (CGA);
- sperm treated with both 1 mM H_2_O_2_ and 100 µM CGA (H_2_O_2_+CGA).

Semen samples were incubated at 37 °C for 1 h (Figure 1) and then progressive motility was evaluated [28]. Then, 100 μL from each aliquot were washed in phosphate buffer saline (PBS) and smeared into slides for use with the acridine orange (AO) test to assess the DNA integrity, while a total of 300 μL of each aliquot was centrifuged at 400× *g* for 15 min. The seminal plasma was stored at −80 °C until the F_2_-IsoP quantification was performed by ELISA and the pellets composed by spermatozoa were used for quantitative real-time polymerase chain reaction (qRT-PCR) to measure Nrf2 mRNA expression.

##### Acridine Orange Test: DNA Integrity Evaluation

The DNA integrity of spermatozoa treated with 1 mM H_2_O_2_, 100 µM CGA, or both 1 mM H_2_O_2_ and 100 µM CGA, and controls was assessed by the AO test, which measures the susceptibility of sperm nuclear DNA to acid-induced denaturation. The test is based on the metachromatic shift of AO fluorescence from green that identifies double-stranded DNA (dsDNA) to red, which means single-stranded DNA (ssDNA), as reported by Tejada et al. [29]. The stock solution of 1% AO (3, 6-bis [dimethylamino] acridine, hemi [zinc chloride] salt; BDH Chemicals Ltd., Poole, UK) was prepared and stored in the dark at 4 °C until use. The staining procedure of the smeared slides was reported in Moretti et al. [30]. A Leitz Aristoplan fluorescence microscope (Leica, Wetzlar, Germany) equipped with a 490 nm excitation light and 530 nm barrier filter was used to evaluate the slides. The green-stained spermatozoa have a double-stranded DNA (dsDNA), while the red/yellow–orange spermatozoa have denatured DNA. For each sample, at least 300 spermatozoa were scored at 1000× magnification and the results are expressed as the percentage of sperm with dsDNA.

##### Sperm RNA Extraction and qRT-PCR for *Nrf2* Expression

Total RNA was extracted from ejaculated spermatozoa using the PureLink^®^ RNA Mini Kit (Thermo Fisher Scientific, Waltham, MA, USA), according to the manufacturer’s instructions. Then, the purity and the concentration of the extracted total RNA were assessed by the A260/A280 absorbance ratios using the Thermo Scientific™ NanoDrop™ One/OneC Microvolume UV–Vis Spectrophotometer (Thermo Fisher Scientific, Waltham, MA, USA).

Complementary DNA (cDNA) was generated from 500 ng of each RNA sample using a High-Capacity cDNA Reverse Transcription Kit (Applied Biosystems, Waltham, MA, USA).

Afterward, the qRT-PCR was performed for mRNA levels of Nrf2 in the spermatozoa. Gene expression was evaluated by a specific PrimePCR™ SYBR™ Green Assay (*NFE2L2* Human #10025636; Bio-Rad Laboratories, Inc., Hercules, CA, USA) using the QuantStudio™ 5 Real-Time PCR System (Thermo Fisher, Waltham, MA, USA). To normalize the mRNA level of Nrf2, a simultaneous mRNA expression profiling of the housekeeping gene glyceraldehyde-3-phosphate dehydrogenase (*GAPDH*; PrimePCR™ SYBR™ Green Assay *GAPDH* Human #10025636; Bio-Rad Laboratories, Inc., Hercules, CA, USA) was also performed in all the analyzed samples. Non-template controls and RT negative samples were run in triplicate as negative controls.

The thermal cycling program was set as follows: UDG (uracil-DNA glycosylases) activation at 50 °C for 2 min and enzyme activation at 95 °C for 2 min, followed by 40 cycles of two steps. The first one was the denaturation at 95 °C for 15 s and the second one at 60 °C for 1 min.

Fluorescent quantitative analysis was determined by the difference of cycle threshold (ΔCt) between the target gene *NFE2L2* (*Nrf2* gene) and the reference gene *GAPDH*. Changes in mRNA levels of Nrf2 were determined using the 2^−ΔΔCt^ method. All qRT-PCR reactions were performed in triplicate.

### 2.4. Statistical Analysis

Statistical analysis was performed with the SPSS version 17.0 for Windows software package (SPSS Inc., Chicago, IL, USA). The normality of the variable distributions was verified with the Kolmogorov–Smirnov test. The Mann–Whitney test was applied to compare the differences between the variables of two groups (step 1). When there were more than two analyzed groups (step 2), first the Kruskal–Wallis test was used to compare the difference among different groups and then a Dunnet post hoc test was applied for pairwise comparisons. Data are reported as median (interquartile range [25th–75th centile], IQR). A *p* value < 0.05 was considered significant.

## 3. Results

### 3.1. Step 1: Effect of CGA on Human Sperm Motility and F_2_-Isoprostane Determination

The semen samples of 66 consecutive patients attending the MVZ Next Fertility Ulm GmbH (Ulm, Germany) were analyzed following the WHO guidelines [28]. A general view of the medians and IQRs of semen parameters of the 66 participants is reported in Table 1.

Each basal semen sample was divided into two aliquots: an untreated control (CTR) and a sample treated with 100 μM CGA (CGA). The percentages of rapid progressive motility and slow progressive motility in the two groups are reported in Table 2. The seminal level of F_2_-IsoPs are reported in Table 2.

After the treatment with CGA, 80.3% (53 out of 66 cases) of the examined samples showed a significant increase in rapid progressive motility percentage (*p* < 0.01; Table 2) and 59.1% (39 out of 66 cases) in slow progressive motility percentage (*p* < 0.01; Table 2).

The levels of F_2_-IsoPs were strongly decreased in all samples supplemented with CGA relative to the controls (*p* < 0.001; Table 2).

In 8 out of 66 considered cases, the rapid and/or slow progressive motility was similar in CGA treated and untreated samples. Spermatozoa from these samples showed some morphological alterations such as high percentages of cytoplasmic residues, head–tail implantation defects, agglutinated spermatozoa (generally associated with immunological problems), and reduced sperm vitality. Nonetheless, CGA still demonstrated a strong antioxidant effect in these cases, as in all other analyzed samples.

### 3.2. Step 2: Effects of CGA on Human Sperm Motility, DNA Integrity, Seminal F_2_-Isoprostane Levels, and Nrf2 mRNA Expression in Samples Treated In Vitro with H_2_O_2_

**Figure 2:**
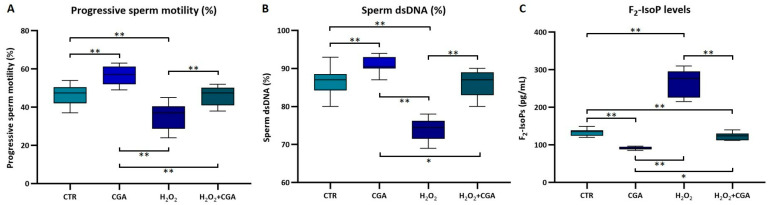
Medians (IQRs) of the percentages of sperm progressive motility (**A**), sperm with dsDNA (**B**), and of F_2_-IsoP levels (**C**) in control samples (CTR), samples treated with 100 μM CGA (CGA), with 1 mM H_2_O_2_ (H_2_O_2_), and with H_2_O_2_+CGA. * *p* < 0.05; ** *p* < 0.01.

In the second step, we used ten samples with normal semen parameters (donors recruited at the Department of Molecular and Developmental Medicine, University of Siena) as reported in the WHO guidelines [28]. Each semen sample was divided into four aliquots: CTR, H_2_O_2_, CGA, and H_2_O_2_+CGA. Then, sperm progressive motility percentage, DNA integrity, F_2_-IsoP concentration, and mRNA levels of Nrf2 were assessed. The treatment with CGA significantly increased, as expected, sperm progressive motility up to 55.5% (IQR: 52.7–59.7%), values that were significantly higher (*p* < 0.01) than that observed in controls (46% [IQR: 40.5–49.2%]), in samples incubated with H_2_O_2_ (35% [IQR: 27.5–38.7%]), and with both H_2_O_2_+CGA (45% [IQR: 39.2–48.0%]). The progressive motility of spermatozoa treated with only H_2_O_2_ was decreased respect to that of control samples (*p* < 0.01); the samples treated with H_2_O_2_+CGA showed a progressive motility similar to that of controls (Figure 2A).

The treatment with CGA significantly increased the percentage of spermatozoa with dsDNA (90.5% [IQR: 90.0–94.1%] with respect to that observed in controls (87% [IQR: 85.5–88.0%]; *p* < 0.01) and in samples incubated with H_2_O_2_+CGA (87.0% [IQR: 83.5–88.8%]; *p* < 0.05). Otherwise, samples treated with H_2_O_2_ (74.5% [IQR: 72.3–75.8%]) showed a significant reduction in the percentage of spermatozoa with dsDNA than that observed in controls, in CGA specimens, and in samples incubated with H_2_O_2_+CGA (*p* < 0.01; Figure 2B).

**Figure 3:**
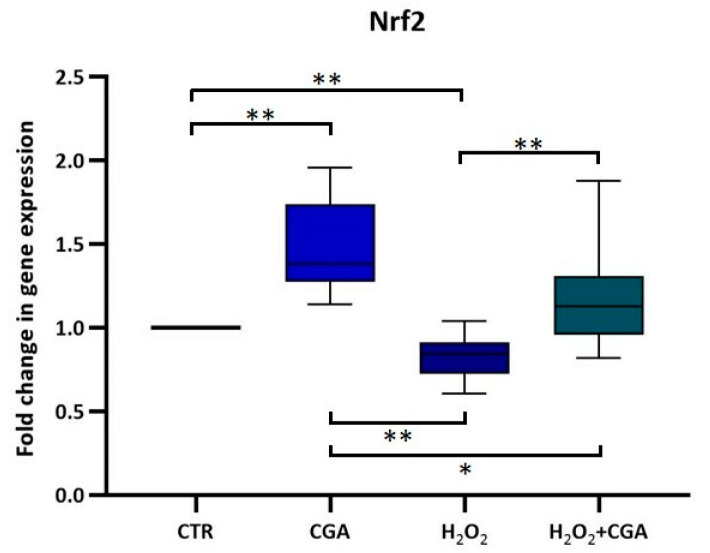
*Nrf2* mRNA expression levels in spermatozoa under different treatments. The graph shows the median (IQR) of the fold change in gene expression. * *p* < 0.05; ** *p* < 0.01.

Finally, samples treated with CGA showed significantly reduced levels of F_2_-IsoPs (90.5 pg/mL [IQR: 88.5 pg/mL–94.1 pg/mL] than that measured in control samples (135.0 pg/mL [IQR: 127.0 pg/mL–136.4 pg/mL]; *p* < 0.01), in samples incubated with H_2_O_2_ (276.4 pg/mL [IQR: 234.2 pg/mL–291.4 pg/mL]; *p* < 0.01), and in samples treated with H_2_O_2_+CGA (124.3 pg/mL [IQR: 114.3 pg/mL–129.3 pg/mL]; *p* < 0.05). The H_2_O_2_+CGA samples also showed lower levels of F_2_-IsoPs than that measured in control and H_2_O_2_ samples (*p* < 0.01), while the treatment with only H_2_O_2_ strongly increased the F_2_-IsoP concentration with respect to that of controls (*p* < 0.01; Figure 2C). The treatment with CGA led to a significant increase in the *Nrf2* expression in spermatozoa compared to the CTR (*p* < 0.01; Figure 3), suggesting the potential role of CGA in the activation of the Nrf2 pathway. The treatment with H_2_O_2_ resulted in a significant reduction in the sperm *Nrf2* expression compared to the control and CGA groups (*p* < 0.01), while the combination of H_2_O_2_+CGA shown a significant recovery of sperm *Nrf2* expression compared to H_2_O_2_ treatment (*p* < 0.01), but still lower than that of CGA treatment alone (*p* < 0.05; Figure 3).

## 4. Discussion

Sperm motility is a critical parameter for male fertility as it directly influences the ability of spermatozoa to reach and fertilize the oocyte. Compromised motility is a hallmark of various reproductive pathologies and is particularly sensitive to OS. ROS-induced damage to mitochondrial function and flagellar structures is a major cause of reduced sperm motility, emphasizing the need for strategies to mitigate OS during semen handling [31]. Indeed, during laboratory manipulation, such as centrifugation and cryopreservation, ROS production often increases, further exacerbating sperm oxidative damage if not controlled by antioxidant systems [32]. It is known that the amount of antioxidants is limited in sperm cells due to the virtual absence of cytoplasm. The seminal plasma plays a crucial role in counteracting ROS because it contains most antioxidant buffering capacity due to enzymatic (e.g., superoxide dismutase, catalase) and non-enzymatic antioxidants (e.g., vitamins, glutathione) able to prevent the OS damaging effects. Human semen is highly heterogeneous and characterized by variability in sperm parameters and antioxidant capacities among individuals, and in infertile subjects, the antioxidant system may be compromised [5,33]. One of the strategies to overcome the problem linked to OS in male infertility is to treat the patients with oral antioxidant supplements with the hope of an improvement of semen parameters. Most studies report a positive relationship between antioxidants and improved male infertility; however, some revealed inconsistent results, highlighting the urgent need for large, randomized, well-designed, placebo-controlled trials [34,35].

The situation is different if the supplementation is given directly to the semen [16] as in the present study. Obviously, in this case, the main purpose is to control ROS production and the damage occurring to the spermatozoa, and therefore motility and vitality are the main parameters protected by antioxidant supplementation in vitro, but it also protects the sperm membrane, DNA and acrosome integrity. Many studies on sperm biology and antioxidant supplementation have focused on selected sperm populations obtained through swim up or density gradient centrifugation techniques. While these methods isolate high-quality spermatozoa, they also exclude the seminal plasma, which serves as the primary antioxidant defense system for sperm cells [10]. The exclusion of seminal plasma in swim up protocols may not accurately reflect the interaction between spermatozoa and their surrounding microenvironment or the efficacy of antioxidant treatments under physiological conditions.

Unlike the study by Noto et al. [14] that focused on CGA’s protective effects in swim up selected spermatozoa, this research aimed to evaluate the effects of CGA supplementation on basal semen samples and under induced OS conditions. By focusing on the basal semen, composed of both seminal plasma and spermatozoa, this study assessed key parameters, including sperm motility, DNA integrity, oxidative biomarkers like F_2_-IsoPs, and the expression of *Nrf2*, a critical regulator of antioxidant defenses. This research included two steps: the first one was carried out at MVZ Next Fertility Ulm GmbH (Ulm, Germany) and the second at the University of Siena, Department of Molecular and Developmental Medicine. In the first step, we observed that 100 μM CGA as a supplement of the basal semen of 66 subjects significantly improved the rapid and slow progressive motility of spermatozoa. This observation could be useful when we need to consider the motility of a basal semen sample in choosing the type of ART, such as in vitro fertilization (IVF) or intracytoplasmic sperm injection (ICSI), in sperm selection processes based on motility such as the swim-up technique, and also in laboratory experiments in basic research. Moreover, CGA supplementation reduced F_2_-IsoP levels, demonstrating its effectiveness in minimizing oxidative damage as a potent antioxidant. F_2_-IsoPs, derived from arachidonic acid, are formed in situ, meaning that they are produced directly where OS occurs, and are then released into the external environment through the action of phospholipase A2. F_2_-IsoP levels measured in seminal plasma and spermatozoa represent a reliable marker of OS for their chemical stability and were measured in different reproductive pathologies linked to male infertility [36]. The findings of this study align with previous research of different groups [37,38,39], which have tested in vitro other antioxidant compounds, alone or in combination, on basal semen from normozoospermic donors. They observed a trend similar to that of CGA in improving or preserving sperm motility while decreasing ROS levels and mitigating OS. In a few samples in our study, the motility did not improve after CGA treatment; these semen samples were characterized by low sperm vitality, abnormal morphology, sperm agglutination or suspected immunological infertility [40], conditions that, obviously, CGA cannot modify. However, also in these cases, as in all other analyzed samples, CGA demonstrated a strong antioxidant effect.

Then, the second step involved an in vitro study in a controlled environment and each basal semen sample was treated with 1 mM H_2_O_2_ to induce OS and CGA was used as an antioxidant. It is noteworthy that the concentration of H_2_O_2_ needed to induce mild OS in basal samples is tenfold higher than that used in free-seminal plasma swim up selected samples by [14]. Therefore, this experiment further demonstrated a potent antioxidant action of CGA since it protects sperm motility, DNA integrity and the plasma membrane in OS conditions. The observed protective action of CGA on DNA is extremely important considering that the integrity of sperm DNA is a critical predictor of outcomes in ART. DNA fragmentation in spermatozoa has been associated with lower fertilization rates, impaired embryo development, and reduced implantation success, increasing the risk of miscarriage [41]. Several studies have shown that interventions aimed at reducing OS and enhancing DNA repair mechanisms can significantly improve ART outcomes [42,43].

Finally, we performed quantitative real time PCR on spermatozoa from the same samples to study the expression of *Nrf2* gene, which encodes for a transcription factor involved in modulating inflammatory processes and playing a key role in male infertility [27]. Despite the limited transcriptional activity of spermatozoa due to its highly compacted chromatin structure, the transcripts can vary with spermiogenesis, capacitation state, different sperm motility and freezing–thawing cycle. Ren et al. [44] concluded their review on this topic with a question: “Are the sperm RNAs which play important roles the remnant of spermatogenesis or the result of sperm transcription? It is not clear at present and needs to be further studied”.

Among different sperm RNAs, the transcription factor Nrf2 is prominent for its involvement in antioxidant defense and protection against OS, since it operates as a master regulator of cellular redox homeostasis by controlling the expression of genes involved in antioxidant pathways. CGA is known to upregulate *Nrf2* expression in other cellular systems [45,46,47] and we observed a similar trend in our samples, suggesting that the CGA antioxidant mechanism of action in human spermatozoa may involve the Nrf2 pathway with a potential impact on sperm progressive motility, and DNA integrity.

A limitation of this research concerns the fact that we did not test the upstream and downstream factors of Nrf2 but just *Nrf2* mRNA expression; other studies in this field are necessary to clearly demonstrate the relationship between CGA and *Nrf2* expression in human spermatozoa. The obtained results seem to demonstrate that human mature spermatozoa possess a residual transcriptional activity, observed also by other authors. For example, Santonastaso et al. [48] reported that curcumin supplementation in freezing medium prevented cryo-damage of human spermatozoa and increased glutathione peroxidase 4 (*GPX4*) gene expression. Also, the addition of melatonin to cryoprotectant during human sperm freezing upregulated the mRNA levels of Nrf2 and other proteins [49]. The investigation of the mRNA expression of enzymatic antioxidants within sperm cells could be interesting to further prove the mechanism of action of CGA.

In conclusion, this study highlights the importance of sperm motility as a sensitive marker of OS-induced damage and emphasizes the advantages of using the basal semen in antioxidant research. CGA demonstrated a strong protective effect in mitigating OS during semen handling and its potential applications in ARTs, both reducing F_2_-IsoPs levels and enhancing sperm motility and DNA integrity. Additionally, our findings contribute to the growing evidence that mature spermatozoa, previously thought to be transcriptionally inert, may exhibit limited but meaningful transcriptional activity. CGA upregulated *Nrf2* expression in spermatozoa exposed to OS, highlighting its role in activating antioxidant pathways and providing new insights into spermatozoa’s capacity to respond to external stressors or environmental changes.

## Figures and Tables

**Table 1:** Medians (IQRs) of age and semen parameters of the 66 patients considered in the first step.

Variables	Median (IQR)
Age	33.0 (29.0–36.0)
Volume (mL)	4.5 (3.8–5.0)
Sperm concentration (10^6^ per mL)	51.5 (38.0–75.0)
Normal morphology (%)	7.0 (5.0–10.0)
Rapid progressive motility (%)	5.5 (3.0–17.5)
Slow progressive motility (%)	34.5 (25.0–40.0)
Vitality (%)	78.0 (67.3–83.8)

**Table 2:** Medians (IQRs) of the percentages of rapid progressive motility and slow progressive motility and F_2_-IsoP levels evaluated in samples treated with 100 μM CGA (CGA) and untreated (CTR).

	CTR	CGA	Statistics
Rapid progressive motility (%)	5.0 (2.3–13.5)	14.0 (7.3–25.0)	*p* < 0.01
Slow progressive motility (%)	31.0 (22.0–40.0)	37.0 (30.0–42.0)	*p* < 0.01
F_2_-IsoPs (pg/mL)	119.5 (95.7–150.6)	10.2 (7.6–14.4)	*p* < 0.001

## Data Availability

The data presented in this study are available on request from the corresponding author. The data are not publicly available due to the privacy of the patients.
